# Treatment of Scarlatina

**Published:** 1850-02

**Authors:** G. W. Brown

**Affiliations:** Port Carbon, Pennsylvania


*Treatment of Scarlatina.* By G. W. Brown, of Port Carbon, Pennsylvania.

To the Editor of the Medical Examiner.

I send you a few notes of my practice in scarlet fever, as it prevailed in this neighborhood in the fall, spring and winter of 1847–'48. I have notes of 126 cases, 21 of which proved fatal. Of the fatal cases, eight died of hydrocephalus; one of hydrocephalus with laryngitis; one of general congestion of the whole venous system; one of ophthalmia, with inflammation of the bowels; one of inflammation of the throat and bronchii; one of abscess of the neck, by which the jugular vein was divided; and three of pericarditis, with general dropsy. One died on the first day, two on the third day, eight on the fourth day, three on the sixth day, one on the tenth day, two on the eleventh day, one on the thirteenth day, one on the fourteenth day, and one on the twenty-second day. Of the secondary affections, dropsy followed in fifteen cases, pericarditis accompanying in six, three of which proved fatal. Rheumatism occurred in six cases, and abscesses in sixteen cases. Four of the cases were adults, and all recovered. Convulsions ushered in a number of cases, but one in particular, in which they lasted for twelve hours, occurring every twenty or thirty minutes. I did not find those cases in which convulsions occurred at the commencement, any more severe or intractable than the others. Those cases in which complete desquamation of cuticle took place recovered most rapidly, and were least frequently followed by secondary affections. Dropsy occurred in the mildest cases; some of them requiring scarce any treatment at all, except an occasional cathartic. In those cases, the affection of the skin being apparently insufficient to eliminate the morbid poison from the system, it fell upon some other organ or tissue of the body. In the commencement of the epidemic I adopted the treatment usually recommended in the books, viz.: an emetic followed by calomel, pepper gargles, laxatives, diaphoretics, tepid sponging and counter-irritation to the throat; but this I soon found to be lamentably deficient, for I lost fully one-half of my cases. About that time, in conversation with Dr. J. S. Carpenter, of Pottsville, I was speaking of the fatal nature of the epidemic, and of the inefficiency of our present mode of treatment, when he suggested a strong solution of the *nitrate of silver* to the throat internally by means of a *probang.* I adopted it immediately, with the most happy results, in all of my cases. Of the last fifty cases treated by it, I scarce lost a patient. I sometimes used a strong solution of the *sulphate of copper*, especially where I desired to vomit the patient at the same time, as was often the case where the throat was filled with shreds of membrane, fœtid matter, &c.; but I think the *nit argent.* preferable in all cases. The strength of the solution was ℈i. to the ℥ of water. I applied the nit. arg. in all my cases as soon as I was called, whether there was ulceration or not, and repeated it once or twice daily till the patient was convalescent. In malignant cases I also used the chloride of soda internally, besides using it as a gargle, and I thought with decided benefit. Blisters, with few exceptions, did no good, but, on the contrary, I thought they did harm by increasing irritation. Lini ments, particularly of iodine, answered better, especially where there was enlargement of the absorbent glands. My treatment then consisted in an emetic at the commencement, followed by calomel in doses of two or three grains every two hours till the bowels were freely moved; then laxatives, to keep up a gentle action on the bowels, sufficient to remove morbid secretions. A mixture of equal parts of the syrup of ipecac. and spir. nit. dulc., to keep a gentle action on the surface, and at any time that it was indicated, pushed sufficiently to produce full vomiting. Nit. arg. to the throat internally, once or twice daily, with gargles of the chloride of soda, and internal administration of the same, when indicated by malignant symptoms. Liniments to the neck, and tepid affusion to the whole body. Cool air and cold applications to the head in the shape of evaporating lotions, so long as the fever continued. I sometimes made use of venesection with decided benefit, but only in vigorous constitutions, and in the very early stage of the disease. Dropsy was treated by bleeding freely and purging, with diuretics and counter-irritation in the latter stages. Pericarditis was treated by the same means as the dropsical effusion, only more promptly. Squill, nitre, digitalis and calomel, in combination, I found to be almost a specific in the second stage of pericarditis, especially if it purged freely.

